# GABA_A_ receptors can initiate the formation of functional inhibitory GABAergic synapses

**DOI:** 10.1111/ejn.12331

**Published:** 2013-08-05

**Authors:** Celine Fuchs, Karine Abitbol, Jemima J Burden, Audrey Mercer, Laura Brown, Jonathan Iball, F Anne Stephenson, Alex M Thomson, Jasmina N Jovanovic

**Affiliations:** 1 UCL School of Pharmacy, University College London29–39 Brunswick Square, London, WC1N 1AX, UK; 2 MRC Laboratory of Molecular Cell Biology, University College LondonLondon, UK

**Keywords:** inhibition, neuroligin-2, postsynaptic, presynaptic, synaptic adhesion, synaptogenesis

## Abstract

The mechanisms that underlie the selection of an inhibitory GABAergic axon's postsynaptic targets and the formation of the first contacts are currently unknown. To determine whether expression of GABA_A_ receptors (GABA_A_Rs) themselves – the essential functional postsynaptic components of GABAergic synapses – can be sufficient to initiate formation of synaptic contacts, a novel co-culture system was devised. In this system, the presynaptic GABAergic axons originated from embryonic rat basal ganglia medium spiny neurones, whereas their most prevalent postsynaptic targets, i.e. α1/β2/γ2-GABA_A_Rs, were expressed constitutively in a stably transfected human embryonic kidney 293 (HEK293) cell line. The first synapse-like contacts in these co-cultures were detected by colocalization of presynaptic and postsynaptic markers within 2 h. The number of contacts reached a plateau at 24 h. These contacts were stable, as assessed by live cell imaging; they were active, as determined by uptake of a fluorescently labelled synaptotagmin vesicle-luminal domain-specific antibody; and they supported spontaneous and action potential-driven postsynaptic GABAergic currents. Ultrastructural analysis confirmed the presence of characteristics typical of active synapses. Synapse formation was not observed with control or *N*-methyl-d-aspartate receptor-expressing HEK293 cells. A prominent increase in synapse formation and strength was observed when neuroligin-2 was co-expressed with GABA_A_Rs, suggesting a cooperative relationship between these proteins. Thus, in addition to fulfilling an essential functional role, postsynaptic GABA_A_Rs can promote the adhesion of inhibitory axons and the development of functional synapses.

## Introduction

GABA_A_ receptors (GABA_A_Rs) are the essential functional postsynaptic components of GABAergic synapses (Schofield *et al*., 1987; Sieghart, 2006; Farrant & Kaila, 2007). Gene deletion studies of some of the most abundant subunits of GABA_A_Rs in mice have demonstrated specific structural changes in inhibitory synapses (Fritschy *et al*., 2012), suggesting that GABA_A_Rs may play a direct role in regulating synapse formation. A number of neuronal adhesion proteins have also been suggested to play a direct structural role in inhibitory GABAergic synapse formation (Sudhof, 2008; Shen & Scheiffele, 2010; Siddiqui & Craig, 2011). Among these, the most prominent role has been attributed to the neuroligin family of postsynaptic adhesion proteins (Scheiffele *et al*., 2000) and their presynaptic binding partners neurexins (Dean *et al*., 2003; Graf *et al*., 2004; Kang *et al*., 2008; Futai *et al*., 2013). The synaptogenic activity of neuroligin-2 (NL2) was first demonstrated in heterologous neuron–HEK293 co-culture systems, in which this protein alone was found to be necessary and sufficient to promote presynaptic differentiation, via neurexin-mediated recruitment of the essential vesicular release machinery (Dean *et al*., 2003; Missler *et al*., 2003; Zhang *et al*., 2005). Surprisingly, gene deletion studies of neuroligins in mice revealed that the density of synaptic contacts was unaltered, suggesting that their presence is not required for the initial formation of synapses (Varoqueaux *et al*., 2006; Poulopoulos *et al*., 2009). However, the prominent functional impairments in GABAergic synaptic transmission in these mice suggest that neuroligins may be required later for consolidation and functional maturation of GABAergic synapses.

The question addressed here was whether GABA_A_Rs alone, under appropriate conditions, could constitute the primary target recognized by inhibitory axons and initiate the formation of synaptic contacts. This would impart selectivity in axonal adhesion to the domains of the plasma membrane enriched in GABA_A_Rs, thereby allowing coordinated consolidation of the structure and the function of synaptic contacts.

To investigate the ability of GABA_A_Rs to promote the formation of contacts with GABAergic neurones, a co-culture model system was developed in which the ‘match’ between the GABAergic neuronal cell type and the postsynaptic GABA_A_R subtype would closely resemble the situation *in vivo*. This ‘matching’ of presynaptic and postsynaptic elements was an attempt to provide the optimal conditions for synapse formation. Accordingly, the neuronal cultures used contained a predominantly GABAergic medium spiny neurone population, which was derived from dissociated embryonic basal ganglia tissue (Ventimiglia & Lindsay, 1998; Goffin *et al*., 2010). The postsynaptic counterparts of medium spiny neurones in these co-cultures were stably transfected human embryonic kidney 293 (HEK293) cells that constitutively expressed α1/β2/γ2-GABA_A_Rs. This GABA_A_R subtype was selected because it was shown to be present in the majority of synapses formed by medium spiny neurons *in vivo* (Gross *et al*., 2011). Synapse formation in this co-culture system was then studied, to assess whether the presence of appropriate GABA_A_Rs is sufficient to promote axon adhesion and formation of functional synapses.

## Materials and methods

### Primary neuronal cultures

Embryonic basal ganglia and hippocampal primary neuronal cultures were prepared as described previously (Ventimiglia & Lindsay, 1998), with minor modifications (Goffin *et al*., 2010). Sprague-Dawley rats (Harlan, UK; the number of pregnant females used was ∼30) were housed and killed according to UK Home Office [and European Communities Council Directive of 24 November 1986 (86/609/EEC)] guidelines. The project was formally approved by the UCL School of Pharmacy Ethics Committee. Basal ganglia regions or hippocampi were dissected from embryonic day 16–17 rat embryos, and dissociated by trituration in Ca^2+^/Mg^2+^-free Hepes-buffered saline solution (Invitrogen). Cells were plated at a density of 30 000 cells/cm^2^ in neurobasal medium containing B27 supplement, glutamine (2 mm), penicillin (100 units), streptomycin (100 μg) and glucose (6 mm) (all from Invitrogen) on glass coverslips or glass-bottomed dishes (MatTek, Ashland, MA, USA) coated with poly-l-lysine (0.1 mg/mL) and laminin (0.01 mg/mL) (both from Sigma-Aldrich). Cultures were incubated in a humidified 37 °C/5% CO_2_ incubator for 12–14 days prior to experimentation.

### Co-cultures

Prior to the formation of co-cultures, control HEK293 cells, or HEK293 cells stably expressing α1β2γ2-GABA_A_Rs (Sanofi-Synthélabo, Paris, France), were transiently transfected with either pCherry or NL2–pCherry, by the use of Effectene reagent (Qiagen). The cells expressing pCherry were referred to as either HEK293 cells or HEK293-GABA_A_R cells. The cells expressing NL2–pCherry were referred to as HEK293-NL2 cells or HEK293-GABA_A_R-NL2 cells. In separate experiments, HEK293 cells were transiently transfected with yellow fluorescent protein (YFP) or with *N*-methyl-d-aspartate (NMDA) receptor NR1–YFP and NR2C subunits, by the use of lipofectamine LTX (Invitrogen) (Chazot *et al*., 1994). These cells were referred to as HEK293-YFP cells or HEK293-NMDAR–YFP cells, respectively. The appropriate HEK293 cells were trypsinized 48 h post-transfection, and added to cultures of medium spiny neurons or hippocampal neurones. The formation of synapses was analysed 2–48 h after plating by electrophysiological recordings and immunolabelling.

### Immunofluorescence

**Figure 1:**
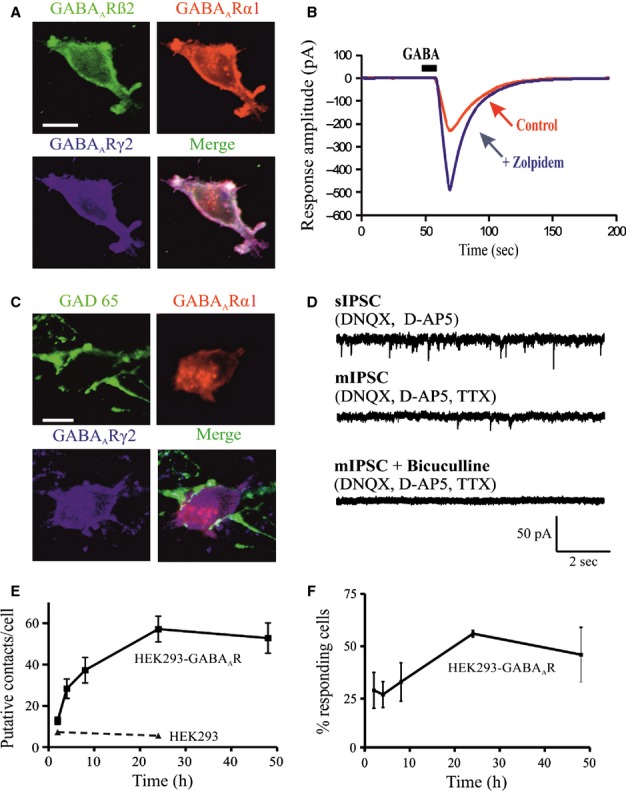
HEK293-GABA_A_R cells expressing functional α1β2γ2-GABA_A_Rs are innervated by embryonic GABAergic medium spiny neurones in co-culture. (A) Cell surface-labelling with antibodies recognizing the extracellular domains of GABA_A_R α1, β2/3 and γ2 subunits in the stably transfected HEK293-GABA_A_R cell line. Scale bar: 20 μm. (B) HEK293 cells responded to GABA (1 μm), puff-applied (10 s) in close proximity, with a large inward current. This response was enhanced by zolpidem (0.4 μm) co-applied in the bathing medium (*n* = 3). (C) Immunolabelling of synapse-like contacts formed between GAD65-positive terminals of GABAergic neurones and postsynaptic α1 and γ2 GABA_A_R subunits expressed in HEK293-GABA_A_R cells. Scale bar: 10 μm. (D) Recordings of sIPSCs in a HEK293-GABA_A_R cell (upper trace) in control medium, and, following TTX application (1 μm), of mIPSCs, in the absence (middle trace) or presence (lower trace) of bicuculline (10 μm). (E) Time course of contact formation. The number of putative contacts per HEK293-GABA_A_R cell at 2, 4, 8, 24 and 48 h, and per control HEK293 cell at 4 and 24 h (mean ± SEM, *n* = 8 in each condition, from two independent experiments). (F) Proportion of HEK293-GABA_A_R cells showing IPSCs increases with time in co-culture to 24 h (mean ± SEM at 2, 4, 8, 24 and 48 h, from two independent experiments).

**Figure 2:**
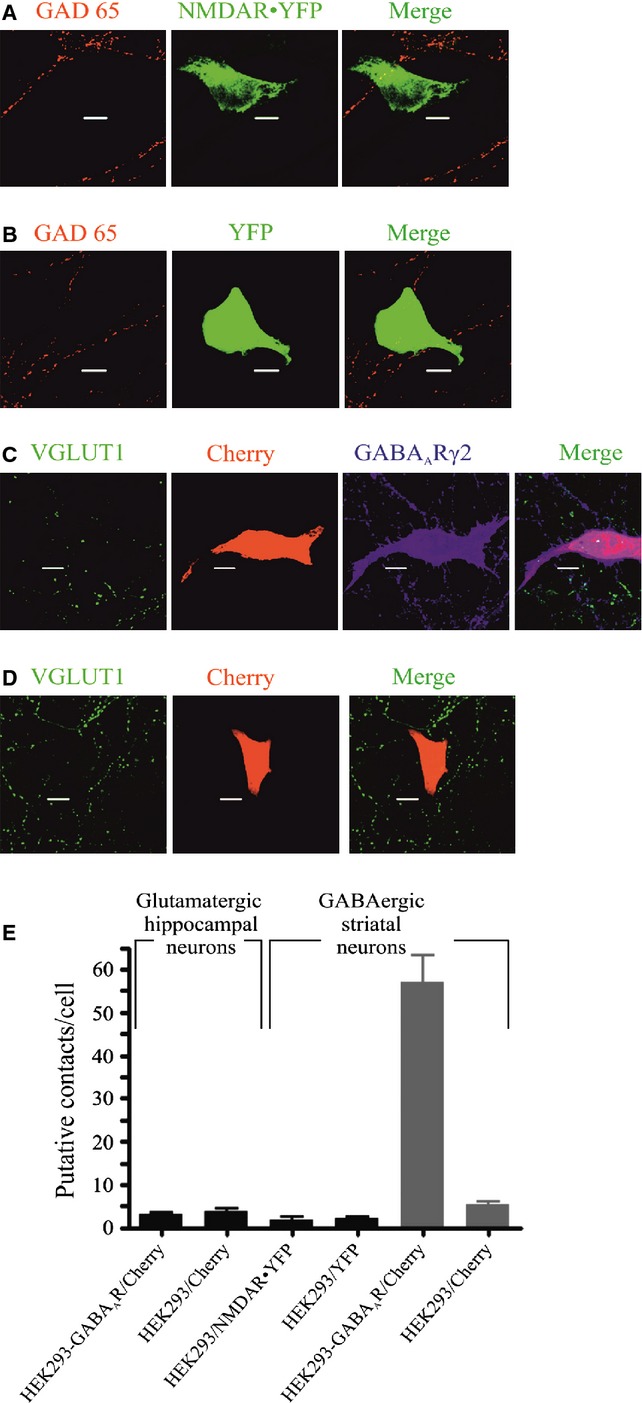
In ‘mismatch’ co-culture experiments, expression of GABA_A_R or NMDA receptor in HEK293 cells failed to promote the formation of contacts with glutamatergic or GABAergic neurones, respectively. (A and B) Immunolabelling of GAD65-positive terminals of GABAergic neurones in co-culture with (A) HEK293-NMDAR–YFP-expressing cells, or (B) HEK293 cells expressing only YFP. Scale bar: 10 μm. (C and D) Immunolabelling of vesicular glutamate transporter 1 (VGLUT1)-positive terminals of hippocampal glutamatergic neurones in co-culture with (C) HEK293-GABA_A_R cells expressing pCherry and GABA_A_Rs (γ2 subunit), or (D) HEK293 cells expressing only pCherry. Scale bar: 10 μm. (E) Quantification of putative contacts formed after 24 h in co-culture between hippocampal glutamatergic neurones and HEK293-GABA_A_R cells or HEK293 cells (mean ± SEM, *n* = 16, from two independent experiments), or between GABArgic neurones and HEK293-NMDAR–YFP cells or HEK293-YFP cells (mean ± SEM, *n* = 16, from two independent experiments), in comparison with the number of contacts formed between GABAergic neurones and HEK293-GABA_A_R cells or HEK293 cells both expressing pCherry (mean ± SEM, *n* = 8, from two independent experiments in each condition).

Cells in co-culture were fixed with 4% paraformaldehyde (PFA)/4% sucrose/phosphate-buffered saline (PBS) for 15 min, washed extensively, and incubated with 1% bovine serum albumin (Sigma-Aldrich)/PBS for 30 min to reduce non-specific binding. Cultures were incubated with rabbit anti-GABA_A_R α1 subunit antibodies (1 : 200, directed against the extracellular α1 N-terminal domain) (Fujiyama *et al*., 2000), mouse anti-β2/3 subunit antibodies (10 μg/mL, directed against the extracellular β2/3 N-terminal domain; MAB341; Merck Millipore, Billerica, MA, USA) or guinea pig anti-γ2 subunit antibodies (1 : 3000, directed against the extracellular γ2 N-terminal domain) (Fritschy & Mohler, 1995) for 14–16 h without permeabilization. Following washing and permeabilization with 0.1% Triton X-100 (Sigma-Aldrich) for 30 min, cultures were incubated with mouse anti-glutamic acid decarboxylase (GAD)65 antibodies (1 : 4000, MAB351; Merck Millipore) for 120 min. Primary antibodies were visualized after incubation with the appropriate goat anti-rabbit, anti-mouse or anti-guinea pig secondary antibodies conjugated to Alexa405, Alexa488, Alexa555, or Cy5 (3 μg/mL; Merck Millipore). The samples were analysed with laser scanning confocal microscopy (Zeiss LSM 510 or 710 Meta) with a × 63 oil-immersion objective. Light levels and detector gain and offset were adjusted to avoid any saturation. Images from at least eight cells from two independent co-cultures, in each of the experimental conditions, were analysed quantitatively. Contacts were identified as regions of colocalization of presynaptic (GAD65), postsynaptic (GABA_A_Rs) or HEK293 cell markers (i.e. pCherry, NL2–pCherry, YFP, or NMDA–YFP) (Figs 1E and 2E; Fig. S1C). The number of contacts between GABAergic axons and HEK293, HEK293-GABA_A_R or HEK293-GABA_A_R-NL2 cells was counted in *z*-series of optical sections (8–10) through a depth of 4–5 μm (Figs 1E and S1C) with lsm 510 software, and the number of contacts with HEK293-YFP or HEK293-NMDAR–YFP cells was counted in *z*-series of 20 sections through a depth of 2–3 μm with lsm 710 software (Fig. 2E). The number of contacts formed between hippocampal glutamatergic axons and HEK293 or HEK293-GABA_A_R cells was counted in *z*-series of 20 sections through a depth of 2–3 μm with lsm 710 software (Fig. 2E).

### Activity-dependent uptake of synaptotagmin antibody

**Figure 3:**
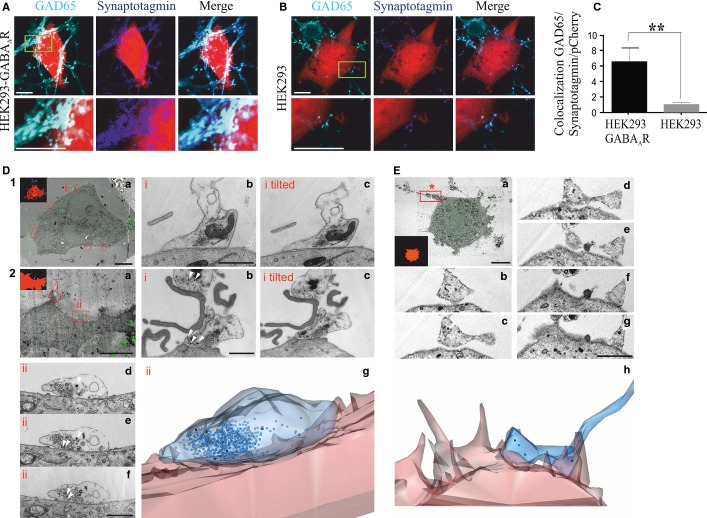
GABA_A_Rs promote the formation of active synaptic contacts. (A and B) Immunolabelling of contacts formed by presynaptic nerve terminals (anti-GAD65), incorporating a vesicle-luminal domain-specific anti-synaptotagmin antibody (Cy5), and the surface of (A) HEK293-GABA_A_R cells, or (B) HEK293 cells expressing only pCherry (scale bar: 10 μm). (C) Quantification of active terminals in contact with the surface of HEK293 cells expressed as colocalization between presynaptic markers and pCherry (arbitrary units; mean ± SEM; HEK293-GABA_A_R,*n* = 16, from two independent experiments; HEK293, *n* = 20, from four independent experiments; **P < 0.05, Student's t-test). (D) Ultrastructure of contacts between neurones and HEK293-GABA_A_R cells labelled and identified (see insert) with anti-synaptotagmin antibodies (Cy3) (scale bar: 10 μm) (D1a and D2a from two independent experiments). Sections of selected contacts (i and ii) are shown in D1b and c and D2b–f. D1b and D1c and D2b and D2c show, in each case, the same section photographed without and with tilt (tilted) (scale bar: 1 μm). The tilt in D2c reveals a second labelled contact on a fine HEK293-GABA_A_R cell process. Serial sections of a single contact are shown in D2d–f, and D2g represents a three-dimensional reconstruction of this contact. Presynaptic vesicles labelled with HRP–diaminobenzidine are indicated by white arrowheads in D2b–f. (E) Ultrastructure of a potential contact with a control HEK293 cell labelled with anti-synaptotagmin antibodies (a, Cy3) (scale bar: 10 μm). Eb–g show serial sections of the selected contact marked with * in Ea (scale bar: 1 μm). Eh represents a three-dimensional reconstruction of this contact. Contacts with control HEK293 cells did not show any structural characteristics typical of synaptic contacts.

To label presynaptic boutons that released synaptic vesicle contents during the incubation period, Cy3-labelled or Cy5-labelled anti-synaptotagmin vesicle-luminal domain-specific antibodies (1 : 50, 105311C3/C5; Synaptic Systems, Goettingen, Germany) were added to co-cultures for 30 min at 37 °C. These antibodies only have access to the luminal domain of synaptotagmin when the vesicle fuses with the plasma membrane and there is continuity between the vesicle lumen and the extracellular space. This occurs specifically in active presynaptic nerve terminals during neurotransmitter release, so these antibodies are used as specific markers of active terminals (Fernandez-Alfonso *et al*., 2006). Co-cultures were washed thoroughly, fixed with 4% PFA/sucrose/PBS, and processed for immunolabelling with anti-GAD65 antibodies, or for electron microscopy. Immunoreactivity was visualized with a Zeiss LSM 710 Meta confocal microscope with a × 63 oil-immersion objective. For each experimental condition, three-colour images of pCherry-positive cells (HEK293-GABA_A_R, *n* = 16 cells, and HEK293-GABA_A_R-NL2, *n* = 14 cells, from two independent co-cultures; and HEK293, *n* = 20 cells, and HEK293-NL2, *n* = 22 cells, from four independent co-cultures) were analysed quantitatively with imagej software (NIH, USA). Briefly, for each cell stack (8–10 sequential *z*-sections of 4–5 μm), the co-localization of GAD65 and synaptotagmin immunolabelling was estimated first, and then compared with pCherry immunolabelling. The labelled area fraction of positive pixels for all three channels was measured, and this value was expressed as the colocalization GAD65/synaptotagmin/pCherry ratio in arbitrary units (Figs 3C and 5C).

### Correlated light and electron microscopy

Co-cultures grown on photo-etched glass-bottomed dishes (MatTek) were fixed immediately, or incubated with horseradish peroxidase (HRP) (10 μg/mL; Sigma-Aldrich) and Cy3-labelled or Cy5-labelled anti-synaptotagmin luminal domain antibodies (1 : 50; 105311C3/C5; Synaptic Systems) prior to fixation with 4% PFA/0.1 m sodium cacodylate buffer. Cells were then imaged (Zeiss LSM 710 Meta) to document the locations of synaptotagmin antibody-positive puncta on HEK293 cells, for later correlation with ultrastructure. Cells were additionally fixed with 2% PFA/1.5% glutaraldehyde/0.1 m sodium cacodylate buffer, and post-fixed with 1% osmium tetroxide/1.5% potassium ferricyanide at 4 °C, and then with 1% tannic acid/0.05 m sodium cacodylate, before being dehydrated and embedded in Epon (TAAB, UK). In HEK293-GABA_A_R cells, HRP taken up during vesicular release was visualized with the HRP/diaminobenzidine reaction (TAAB) prior to osmication. Previously identified HEK293 cells were located on the block face, and 70-nm serial sections were collected on Formvar-coated slot grids, post-stained with lead citrate, and imaged with an FEI Tecnai 20 microscope (FEI, Hillsboro, OR, USA), equipped with an Olympus-SIS Morada CCD camera (Olympus, Tokyo, Japan) and item software, or with a CM 120 Bio-Twin microscope (Philips). Contacts were reconstructed in three dimensions by combining ultrastructural data from 10–19 70-nm serial sections by the use of reconstruct software (Fiala, 2005).

### Time-lapse imaging

Cultured medium spiny neurones (7 days *in vitro*) were transfected with pEGFP (Clontech), by the use of magnetofection (OZ Biosciences, Marseille, France), as described previously (Buerli *et al*., 2007). At 12–13 days *in vitro*, HEK293-GABA_A_R cells transfected with pCherry were added to the neurones. Time-lapse recording was performed 24 h later (Zeiss LSM 710 Meta), with serial images being taken (× 20 objective) every minute for 120 min.

### Immunoblotting

Cultures of HEK293, HEK293-GABA_A_R or HEK293-GABA_A_R-NL2 cells were washed with PBS and lysed with pre-warmed 2% sodium dodecyl sulphate. The protein concentration was estimated with the bicinchoninic acid assay (Thermo Scientific), and the same amount of total protein (200 μg/lane) was subjected to sodium dodecyl sulphate–polyacrylamide gel electrophoresis with 8% gels. Immunoblotting was carried out with primary rabbit anti-NL2-specific antibodies (1 : 1000 dilution, 129 202; Synaptic Systems) and secondary alkaline phosphatase-conjugated anti-rabbit antibodies (1 : 2000; Jackson ImmunoResearch, West Grove, PA, USA), with detection with nitroblue tetrazolium/5-bromo-4-chloro-3′-indolyphosphate substrate (Promega, Madison, WI, USA).

### Electrophysiology

**Figure 4:**
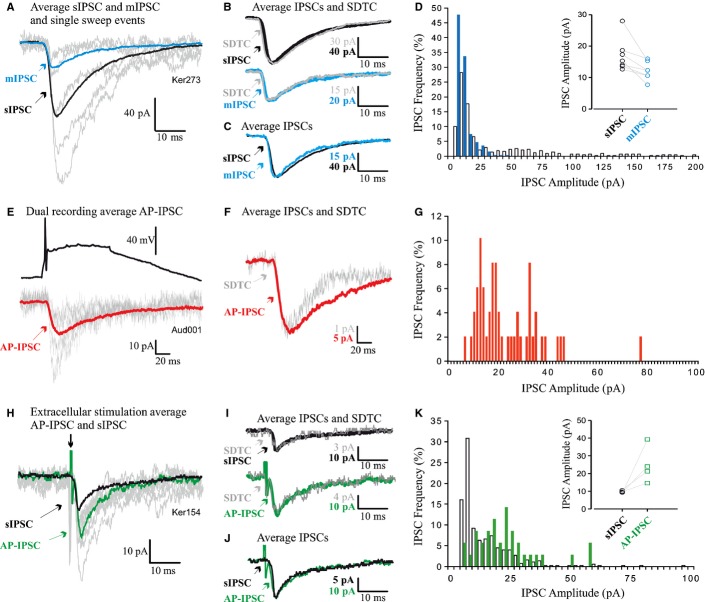
GABA_A_Rs support the formation of functional synapses. (A–D, E–G, and H–K) IPSCs in three HEK293-GABA_A_R cells. (A, E, and H) Averaged sIPSCs and mIPSCs (A) and AP-IPSCs (E and H) superimposed on single-sweep sIPSCs demonstrate the event-to-event fluctuations in amplitude typical of connections that include multiple release sites. (B, F, and I) Averaged IPSC and SDTC scaled and superimposed demonstrate that the single-sweep IPSCs included in averages were of similar shape, and (for E and H) of similar onset latency. (C) Averaged sIPSC and mIPSC and (J) averaged stimulus-elicited AP-IPSC and sIPSC, scaled and superimposed to compare time courses, demonstrate that all sIPSCs, mIPSCs and AP-IPSCs in each HEK293-GABA_A_R cell were similar in shape. (D, G, and K) IPSC amplitude distributions for mIPSCs and sIPSCs (D), AP-IPSCs recorded during dual recordings (G), and sIPSCs and AP-IPSCs (stimulus-elicited) (K). sIPSC and mIPSC amplitudes in the same HEK293-GABA_A_R cells are compared in D, and sIPSCs and AP-IPSCs in the same cell are compared in K. Inserts compare median IPSC amplitudes for seven (D) and four (K) HEK293-GABA_A_R cells.

HEK293-GABA_A_R or HEK293-GABA_A_R-NL2 cells were identified by their fluorescence (X-Cite series 120Q light source; EXFO) and recorded in visually guided whole-cell mode with infrared differential interference contrast optics (Olympus BX51). The extracellular medium contained 130 mm NaCl, 4 mm KCl, 10 mm Hepes, 20 mm NaHCO_3_, 10 mm glucose, 1 mm MgCl_2_, and 2 mm CaCl_2_, and was equilibrated with 5% CO_2_/95% O_2_ (pH 7.4; 330 mosmol/L; flow rate, 1.8 mL/min). All recordings of spontaneous inhibitory postsynaptic currents (IPSCs) [sIPSCs, which include both miniature IPSCs (mIPSCs) and action potential (AP)-driven IPSCs (AP-IPSCs)], mIPSCs and AP-IPSCs in HEK293-GABA_A_R-NL2 cells, all recordings of sIPSCs, mIPSCs, stimulus-elicited AP-IPSCs and half of the AP-IPSCs recorded in dual recordings in HEK293-GABA_A_R cells were made at 32 °C, whereas 67 of the 125 dual whole-cell recordings with HEK293-GABA_A_R cells (Figs 4E–G) were made at room temperature. Patch pipettes had a final resistance of 3–8 MΩ when filled with an intracellular solution containing 130 mm KCl, 3 mm NaCl, 4.5 mm phosphocreatine, 10 mm Hepes, 1 mm EGTA, 3.5 mm Na-ATP, 0.45 mm Na-GTP, and 2 mm MgCl_2_ (adjusted to pH 7.2 with KOH, 290–300 mosmol/L). To test the responsiveness of the HEK293 cells to GABA and to investigate the pharmacological properties of the expressed receptors, GABA (1 μm; Tocris Bioscience, Bristol, UK) was puffer-applied, and zolpidem [*N*,*N*-dimethyl-2-(6-methyl-2-*p*-tolylimidazo[1,2-a]pyridin-3-yl)acetamide, 0.4 μm; Sigma/RBI] was added to the bathing medium. Zolpidem is a GABA_A_R benzodiazepine site agonist with a higher affinity for α1 subunit-containing GABA_A_Rs than for α2-containing and α3-containing receptors, and very low affinity for α5-containing receptors (α4-containing and α6-containing receptors are insensitive to benzodiazepines). Given that the responsiveness to zolpidem is dependent on the presence of a γ2 subunit and surface expression is determined by the inclusion of a β subunit, we can conclude that enhancement of the GABA response by zolpidem demonstrates that the GABA_A_Rs expressed at the surface of HEK293-GABA_A_R cells were predominantly α1β2γ2-GABA_A_Rs.

sIPSCs, mIPSCs and AP-IPSCs were recorded at a membrane potential of −60 mV (MultiClamp 700B, with series resistance compensation; Molecular Devices), and, in preliminary experiments, in the presence of 20 μm 6,7-dinitroquinoxaline-2,3-dione (DNQX, first dissolved in dimethyl sulphoxide; Tocris Bioscience) and 50 μm d-2-amino-5-phosphonovalerate (d-AP5) (Abcam Biochemicals, Cambridge, UK). As neither d-AP5 nor DNQX influenced HEK293 cell properties or spontaneous postsynaptic currents in these cells, subsequent experiments were performed without these blockers. mIPSCs were recorded in the presence of 1 μm tetrodotoxin citrate (TTX) (Abcam Biochemicals) (Fig. 1D; Fig. S1B). In some of these recordings, the GABA_A_R antagonist bicuculline methochloride (10 μm; Abcam Biochemicals) was also added (Fig. 1D; Fig. S1B), to determine whether these spontaneous miniature events were mediated by GABA_A_Rs. IPSC frequencies were obtained from periods of continuous recording of not < 5 min. These frequencies are expressed as the number of events per second that exceeded a current threshold (2 × noise) and resembled averaged IPSCs in shape.

### Dual whole-cell recordings

Presynaptic medium spiny neurones were recorded in current-clamp mode, and presynaptic APs were elicited by injecting depolarizing current. Responses in neighbouring HEK293 cells were recorded in voltage-clamp mode. If a test produced no response in the simultaneously recorded HEK293 cell, both electrodes were withdrawn and two new cells were ‘patched’. Extracellular stimulation of neurones was performed with a patch electrode containing extracellular medium. This electrode was positioned close to a neurone, the neurone was stimulated to elicit APs, and, if the test was negative, the electrode was moved to appose another neurone. The axons of these neurones often formed bundles in culture. Extracellular stimulation may therefore have activated several axons, accounting for the large IPSCs elicited by stimulation in some cultures and their longer time course than those of sIPSCs and the AP-IPSCs recorded during dual recordings, when the output of only one neurone was activated and recorded.

### Data acquisition and analysis

Continuous electrophysiological recordings were filtered at 5 kHz, digitized at 10 kHz (CED 1401; Cambridge Electronic Design) and collected with spike2 (Cambridge Electronic Design). Putative postsynaptic events were detected according to a current threshold, and selected (manually, by shape) before offline analysis (MSpike, D.C. West, UCL School of Pharmacy). Access resistance was monitored, and recordings were discarded if it exceeded 15 MΩ. For computed averages of mIPSCs, sIPSCs, and AP-IPSCs, the fast rising phase of the IPSC, the fast rising phase of the presynaptic AP (dual recordings) or the extracellular stimulus was used, respectively, as a trigger. The shape of the averaged IPSC and the timing of its peak informed individual manual IPSC amplitude measurements. The standard deviation time course (SDTC), which plots the standard deviation about the mean of the averaged IPSC, was computed in parallel with each average, prior to further analysis, to ensure that events included in averages were of similar shape. When events with different shapes, or different latencies, are included in an average, the peak of the SDTC does not coincide with the peak of the average, indicating variation in the onset latency or the rising and/or falling phase of the events. The IPSC 10–90% rise time (RT, the time taken for the IPSC to rise from 10% to 90% of its peak amplitude) and width at half amplitude (HW) were measured from averages, and amplitude distributions were constructed from single event measurements. As it is possible that very high sIPSC frequencies could have obscured the falling phase of the averaged IPSC, averages of a subset of records, selected as being devoid of such spontaneous events, were used to measure the IPSC HW. However, the median IPSC amplitude was calculated from measures of the entire population of detected synaptic events for each cell, and amplitude distributions contained all events. Data are given as mean ± standard error of the mean (SEM), and Student's *t*-test (e.g. for sIPCS or mIPSC frequencies and IPSC time course parameters) was used to test for significant differences between populations. For skewed distributions, however, median values plus the 25th and 75th percentiles are given. sIPSC, mIPSC and AP-IPSC amplitude distributions were first tested for normality (Shapiro–Wilk test), and, because the majority were found not to be normally distributed, a Mann–Whitney *U*-test was used for unpaired comparisons (e.g. sIPSC or mIPSC amplitudes in HEK293-GABA_A_R cells vs. sIPSC or mIPSC amplitudes in HEK293-GABA_A_R-NL2 cells), and a Wilcoxon test was used for paired comparisons (e.g. sIPSC amplitudes vs. mIPSC amplitudes, and sIPSC amplitudes vs. AP-IPSC amplitudes). In the figures, some electrophysiological traces were filtered to reduce high-frequency noise (three-point running average), and stimulus artefacts were reduced graphically, for clarity. psi-plot (Poly Software International), graphpad prism (GraphPad Software) and Excel (Microsoft) were used for analysis, plotting, and statistical tests.

## Results

### Postsynaptic GABA_A_Rs mediate adhesion of GABAergic axons

To test whether GABA_A_Rs can play a direct role in synaptic target recognition, a novel co-culture model system was established. This consisted of a homogeneous population of GABAergic basal ganglia medium spiny neurones co-cultured with HEK293 cells stably expressing α1/β2/γ2-GABA_A_Rs (HEK293-GABA_A_R; Fig. 1A), the most prevalent postsynaptic receptor subtype present in synapses of these neurons (Gross *et al*., 2011).

HEK293-GABA_A_R cells responded to GABA (1 μm), puff-applied (10 s) in close proximity, with a large inward current (−518 ± 24.2 pA, *n* = 9). This response was enhanced by the α1 subunit-preferring GABA_A_R benzodiazepine site agonist zolpidem (0.4 μm) co-applied in the bathing medium (58 ± 6.4% enhancement, *n* = 3), confirming the expression of functional α1/β2/γ2-GABA_A_Rs in this cell line with the expected pharmacological response (Fig. 1B).

The presence of functional α1/β2/γ2-GABA_A_Rs at the plasma membrane was sufficient to initiate adhesion of GAD65-positive presynaptic terminals and the formation of contacts between neurones and HEK293 cells, as early as 2 h after plating (Fig. 1E). The number of putative synaptic contacts per HEK293 cell increased rapidly, reaching 57.1 ± 6.2 in HEK293-GABA_A_R cells at 24 h (mean ± SEM, *n* = 8; Fig. 1E). Electrophysiological recordings showed that the proportion of HEK293-GABA_A_R cells in co-culture that showed sIPSCs (Fig. 1D) also reached a maximum at 24 h (56%; Fig. 1F). This correlated well with the time scale of putative contact formation (Fig. 1E). The smaller, TTX-resistant mIPSCs, recorded when APs were blocked, were abolished by the GABA antagonist bicuculline (10 μm,*n* = 6; Fig. 1D), indicating their mediation by GABA release from the medium spiny neurones.

This process of rapid contact formation between neurones and HEK293-GABA_A_R cells was not observed in experiments with control HEK293 cells (Figs 1E, 2B, and 2E). This was also the case with ‘mismatch’ experiments, when HEK293 cells expressing NMDA receptors were co-cultured with GABAergic medium spiny neurones (Figs 2A and E), or when HEK293-GABA_A_R cells (Fig. 2C) or control HEK293 cells (Fig. 2D) were co-cultured with glutamatergic hippocampal neurones (Fig. 2E). Thus, the expression of ‘mismatched’ receptors in HEK293 cells was not sufficient to promote contact formation in co-cultures with GABAergic medium spiny neurones or glutamatergic hippocampal neurones.

### GABA_A_Rs initiate the formation of functional synaptic contacts

Many of the contacts between medium spiny neurones and HEK293-GABA_A_R cells showed activity-dependent uptake of fluorescently labelled anti-synaptotagmin synaptic vesicle-luminal domain-specific antibodies (Fig. 3A). Co-localization between GAD65-positive and luminal synaptotagmin-positive terminals that formed contacts with the surface of HEK293-GABA_A_R cells (Fig. 3A) or control HEK293 cells (Fig. 3B) was quantified with imagej. This analysis demonstrated that the number of active contacts formed with HEK293-GABA_A_R cells was significantly greater than the number of contacts formed with control HEK293 cells (means ± SEM: 6.5 ± 1.7 arbitrary units, *n* = 11, vs. 1.01 ± 0.28 arbitrary units, *n* = 18; Student's *t*-test, *P* = 0.015; Fig. 3C). Ultrastructural analysis of synaptotagmin-positive contacts between neurones and HEK293-GABA_A_R cells revealed characteristics typical of active synapses (Figs 3D1 and D2). These included a region of close membrane apposition between presynaptic and postsynaptic elements, and multiple membrane-bound vesicles and mitochondria in the same, or adjacent, sections. Dark, diaminobenzidine-positive synaptic vesicles observed within the contacts formed with HEK293-GABA_A_R cells confirmed the activity-dependent incorporation of HRP (Fig. 3D2). This demonstrates that, during the incubation, prior to fixation, the lumens of some synaptic vesicles were in continuity with the extracellular space; that is, these vesicles had undergone exocytosis and neurotransmitter release. These characteristics were not observed in rare contacts formed between neurones and control HEK293 cells (Fig. 3E). Time-lapse confocal imaging demonstrated that contacts formed between neurones and HEK293-GABA_A_R cells were stable over a time period of 120 min (Movie S1).

### Recordings of inhibitory postsynaptic potentials in HEK293-GABA_A_R cells

Electrophysiological recordings were made from HEK293-GABA_A_R cells to determine whether the putative contacts identified with immunolabelling and electron microscopy could support synaptic activity. After 22–26 h in co-culture, HEK293-GABA_A_R cells were identified by fluorescence and recorded in whole-cell mode. In these recordings, sIPSCs were detected at a frequency of 2.5 ± 0.75/s (mean ± SEM, *n* = 20; Figs 1D and 4A–C). In contrast, in unmodified HEK293 cells (*n* = 10) and control HEK293 cells (expressing only pCherry, *n* = 10), no synaptic events were recorded (data not shown). sIPSC amplitude distributions were skewed, with a large population of small-amplitude events, followed by a ‘tail’, or one or more discrete peaks of larger events (Fig. 4D) (median sIPSC amplitude, 15.3 pA; 25–75%, 13.7–18.7 pA; *n* = 7). These larger events were blocked by TTX (1 μm; Figs 1D and 4A–D), suggesting that these larger spontaneous events represented AP-driven release of GABA, and amplitude distributions became more discrete (compare blue with white bars in the histogram shown in Fig. 4D; median mIPSC amplitude, 12.3 pA; 25–75%, 10.7–15.3 pA). This demonstrates that spontaneous synaptic events recorded under control conditions included a population of larger events that were dependent on APs.

In some HEK293-GABA_A_R cells, sIPSC frequency estimates were compromised by the ability of large spontaneous events to obscure the smaller events when they overlapped in time. The frequencies of sIPSCs and mIPSCs cannot, therefore, be directly compared for all seven HEK293-GABA_A_R cells treated with TTX, but in three such cells in which events could be distinguished satisfactorily, the sIPSC frequency was 8.973 ± 1.66/s (mean ± SEM), and the mIPSC frequency was 4.69 ± 2.07/s. For all seven cells subsequently treated with TTX, the mean sIPSC frequency was 5.5 ± 1.58/s and the mean mIPSC frequency was 4.23 ± 1.25/s. These sIPSC frequencies are higher than the average for the larger population given above, which includes cells not treated with TTX, because those selected for mIPSC analysis were those with the higher spontaneous frequencies.

### Paired whole-cell recordings

To confirm that these synapse-like contacts could indeed support AP-driven GABA release (AP-IPSCs), paired whole-cell recordings were performed for presynaptic basal ganglia medium spiny neurones and neighbouring HEK293-GABA_A_R cells (Figs 4E–G). The proportion of such paired recordings that revealed a connection was 1 : 125. An event of the appropriate shape that follows each presynaptic AP at fixed latency can be assumed to be the result of the synchronous release of transmitter in response to that AP, i.e. AP-driven release. Despite their similarity in shape and onset latency shape, however, AP-IPSCs fluctuated in amplitude from event to event, because several synaptic contacts typically contribute to each synaptic connection between two cells, and the release of transmitter is stochastic. This fluctuation can be seen in Figs 4E and H, in which an averaged AP-IPSC is superimposed on several of the single-sweep AP-IPSCs that contributed to the average. That the shapes and onset latencies of all events included in the averaged AP-IPSCs were similar is indicated by the shape of the SDTC, which matches the shape of the average (Figs 4B, F, and I). To increase test numbers, extracellular stimulation was also employed (Fig. 4H–K). These AP-IPSCs, whether from dual recordings or extracellular stimulation, were generally of similar duration to sIPSCs in the same HEK293-GABA_A_R cells. This can be seen in Fig. 4J, in which the averaged IPSCs shown in Fig. 4H (sIPSC and AP-IPSC) are scaled to match amplitudes and superimposed. This is also demonstrated in Figs 7A and B, which compare the RTs and HWs of these IPSCs. Like sIPSCs (Fig. 4D), AP-IPSCs were larger than mIPSCs (23.32 pA for the dual recording AP-IPSC and 24.83 ± 5.22 pA, *n* = 4, for the stimulus-elicited AP-IPSCs) (Fig. 4; compare mIPSC mean amplitudes, insert in D, with AP-IPSC amplitude distributions in G and K), suggesting that several synapse-like contacts from one axon contributed to each AP-IPSC.

### GABA_A_Rs expressed with NL2 promote further synaptogenesis

The experiments described above demonstrate that the presence of only one type of neuronal GABA_A_R expressed stably in HEK293 cells can promote the adhesion, formation and stabilization of functional presynaptic GABAergic axon terminals (Figs 4). This process occurs independently of the neuronal adhesion protein NL2, as demonstrated by the lack of expression of this protein in HEK293-GABA_A_R cells by immunoblotting (data not shown). In the light of previously published evidence for an equivalent role for NL2 in synapse assembly, NL2–pCherry was transiently expressed in HEK293-GABA_A_R cells (HEK293-GABA_A_R-NL2; Fig. S1A) to determine whether the co-expression of NL2 with α1/β2/γ2-GABA_A_Rs may further promote synapse formation and maturation in our co-cultures. Contacts between the basal ganglia medium spiny neurones and HEK293-GABA_A_R-NL2 cells formed rapidly, reaching 76.2 ± 12.1 per cell at 24 h (mean ± SEM, *n* = 8; Fig. S1C). The timing of contact formation paralleled the detection of synaptic GABAergic currents (Fig. S1B and D).

**Figure 5:**
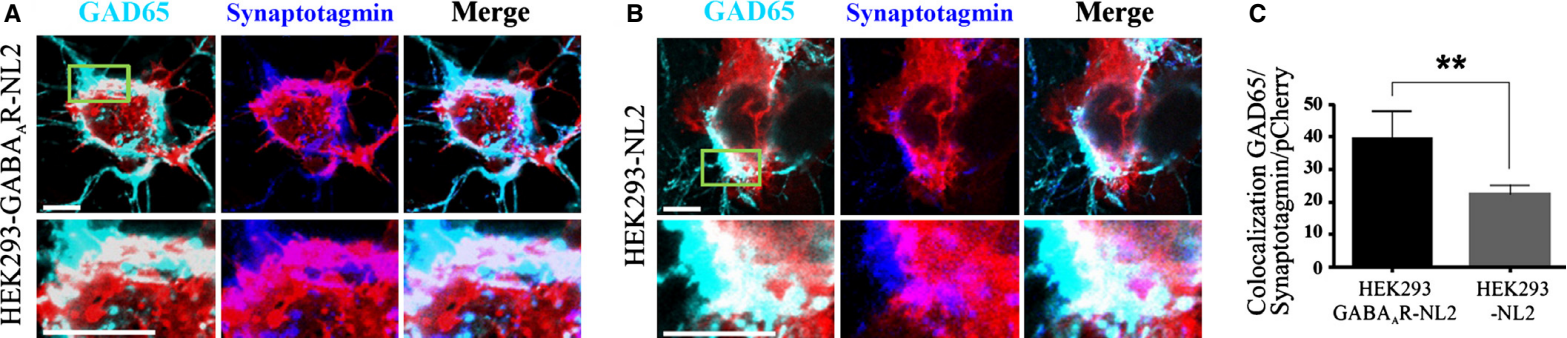
GABA_A_Rs expressed with NL2 promote further synaptogenesis. (A and B) Immunolabelling of contacts between presynaptic nerve terminals (anti-GAD65), incorporating a luminal domain-specific anti-synaptotagmin antibody (Cy5) and the surface of HEK293-GABA_A_R-NL2 cells (A) or of HEK293-NL2 cells (B) (NL2–pCherry) (scale bar: 10 μm). (C) Quantification of active terminals at the surface of HEK293-GABA_A_R-NL2 or of HEK293-NL2 cells, expressed as colocalization between presynaptic markers and postsynaptic NL2–pCherry (arbitrary units; mean ± SEM; HEK293-GABA_A_R-NL2, *n* = 14 from two independent experiments; HEK293-NL2, *n* = 22 from four independent experiments; **P < 0.05, Student's t-test).

The majority of contacts formed with HEK293-GABA_A_R-NL2 or HEK293-NL2 cells incorporated the fluorescently labelled anti-synaptotagmin vesicle-luminal domain-specific antibodies (Figs 5A and B). Quantification revealed that the innervation of HEK293-GABA_A_R-NL2 cells (mean ± SEM, 39.2 ± 8.7 arbitrary units, *n* = 15) was significantly greater than that of HEK293-NL2 cells (mean ± SEM, 22.4 ± 2.7 arbitrary units, *n* = 18; Student's *t*-test, *P* = 0.03; Fig. 5C), or of HEK293-GABA_A_R cells (mean ± SEM, 6.5 ± 1.7 arbitrary units, *n* = 11; Student's *t*-test, *P* = 0.001; Fig. 3C). Ultrastructural analysis of contacts formed between medium spiny neurones and HEK293-GABA_A_R-NL2 or HEK293-NL2 cells also demonstrated close membrane appositions between presynaptic and postsynaptic elements, multiple membrane-bound vesicles, and mitochondria, i.e. structural characteristics of mature synapses (Figs S2A and B).

### Co-expression of GABA_A_Rs and NL2 enhances synaptic efficacy

**Figure 6:**
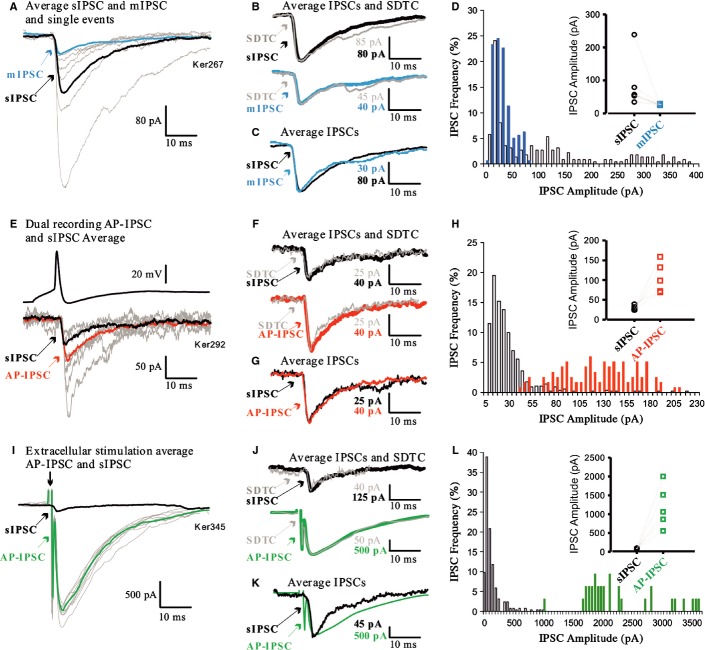
Co-expression of GABA_A_Rs and NL2 enhances synaptic efficacy. IPSCs in three HEK293-GABA_A_R-NL2 cells (A–D, E–H, and I–L). (A, E, and I). Averaged sIPSCs and mIPSCs (A), and averaged AP-IPSCs (dual recordings vs. extracellular stimulation) and sIPSCs (E and I), superimposed on single-sweep sIPSCs. (B, F, and J) Averaged IPSC and SDTC scaled and superimposed demonstrates the similarity in shape of events included in the averages. (C) Averaged sIPSC and mIPSC, (G) averaged sIPSC and AP-IPSC (dual recording), and (K) averaged AP-IPSC (stimulus-elicited) and sIPSC, scaled and superimposed to compare time courses. (D, H, and L). IPSC amplitude distributions to compare the amplitudes of sIPSCs and mIPSCs (D) and of AP-IPSCs and sIPSCs (H and L) in the same cells. Inserts compare median IPSC amplitudes for seven (D), five (H) and five (L) cells.

**Figure 7:**
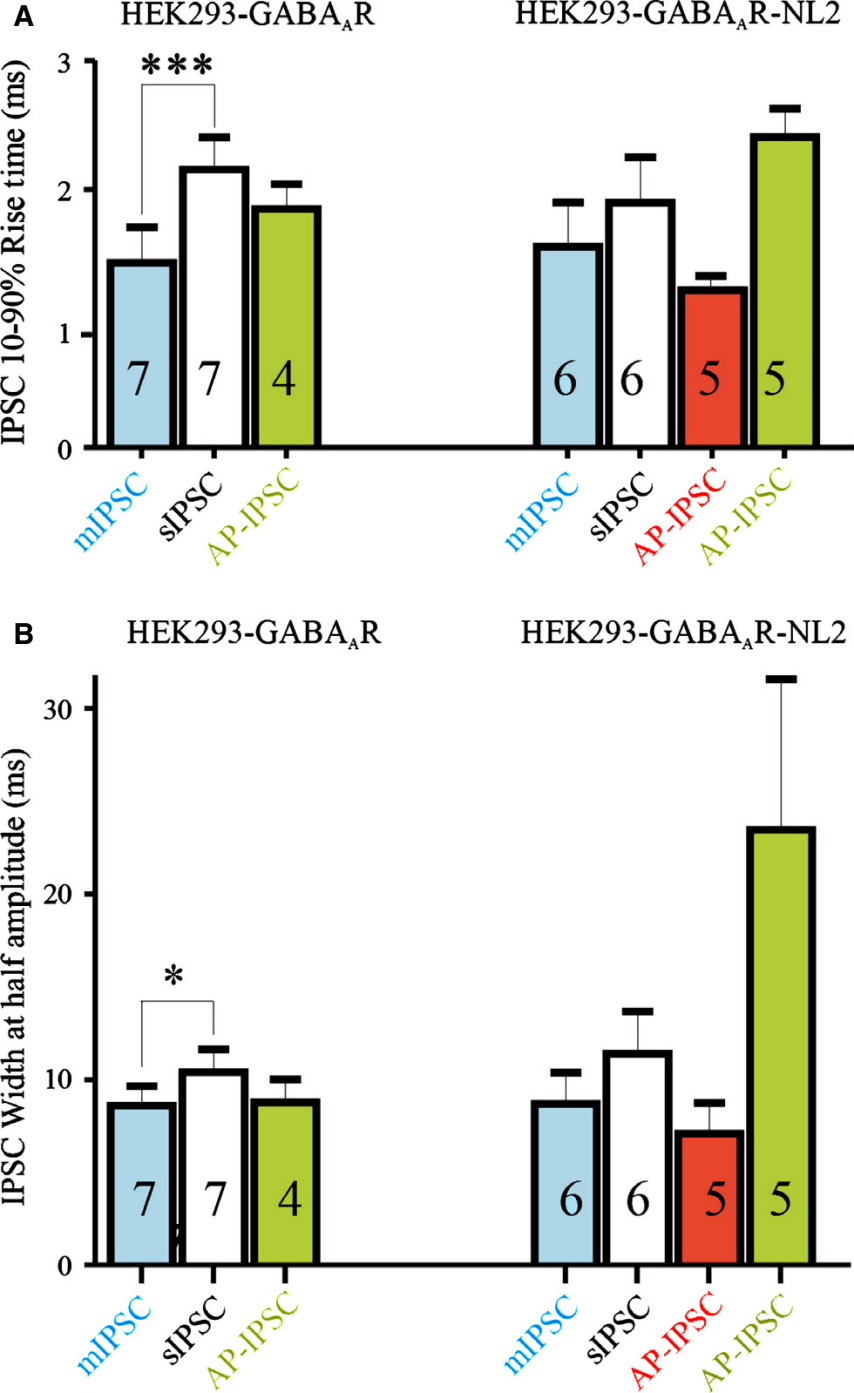
Time course of IPSCs: properties of mIPSCs, sIPSCs, dual recording AP-IPSCs and stimulus-elicited AP-IPSCs (means ± SEM) in HEK293-GABA_A_R cells (left) and HEK293-GABA_A_R-NL2 cells (right). (A) Comparison of the RTs. (B) Comparison of the HWs. Asterisks indicate where two populations differed significantly from each other (*P < 0.05, ***P < 0.001, one-way anova). The numbers in the bars indicate the number of cells included in each population. Time course parameters for AP-IPSCs (dual recordings) without NL2 are not shown, as one of the two examples of these AP-IPSCs was recorded at room temperature and is not therefore comparable.

Electrophysiological recordings were made from HEK293-GABA_A_R-NL2 cells to determine the functional properties of putative synaptic contacts formed between these cells and medium spiny neurones in co-culture. In these recordings, a high sIPSC frequency was observed (mean ± SEM, 12.8 ± 2.96/s, *n* = 16; Fig. S1B; Figs 6A–C). In contrast, no synaptic events were observed when recording**s** were made from HEK293-NL2 cells (*n* = 6). As in HEK293-GABA_A_R cells, sIPSCs in HEK293-GABA_A_R-NL2 cells fluctuated in amplitude, and were larger than mIPSCs in the same cells (Figs 6A and D), but were of similar shape (Figs 6C and 7). In 10 paired recordings, APs in five medium spiny neurones elicited AP-IPSCs in a neighbouring, simultaneously recorded HEK293-GABA_A_R-NL2 cell (Figs 6E–G) (mean amplitude, 106.14 ± 17.33 pA). This 1 : 2 ‘hit rate’ is considerably higher than that seen in HEK293-GABA_A_R cells (1 : 125). Extracellular stimulation was also employed (Figs 6I–K) (hit rates of 5 : 8 in HEK293-GABA_A_R-NL2 cells vs. 4 : 46 in HEK293-GABA_A_R cells; mean amplitude, 1187.2 ± 253.68 pA). The dual recording AP-IPSCs were generally of similar duration to sIPSCs in the same HEK293 cells (Figs 6G and K) and larger than mIPSCs in similar cells (Fig. 6; compare D with H and L), suggesting, again, that several synapse-like contacts from one axon contributed to each AP-IPSC. The much larger amplitudes of the stimulus-elicited AP-IPSCs in HEK293-GABA_A_R-NL2 cells suggest the activation of several connected axons.

Overall, the shape and time course of IPSCs recorded in HEK293-GABA_A_R and HEK293-GABA_A_R-NL2 cells were similar (Fig. 7). In HEK293-GABA_A_R cells, the RTs of sIPSCs and of mIPSCs (2.2 ± 0.25 ms and 1.5 ± 0.25 ms, respectively; Fig. 7A) and the HWs (10.4 ± 1.23 ms and 8.5 ± 1.09 ms, respectively, *n* = 7; Fig. 7B) were similar to those in HEK293-GABA_A_R-NL2 cells (sIPSC RT, 1.9 ± 0.35 ms; mIPSC RT, 1.5 ± 0.35 ms; sIPSC HW, 11.4 ± 2.25 ms; mIPSC HW, 8.7 ± 1.69 ms; *n* = 6). The briefer time course of mIPSCs than of sIPSCs was apparent in both conditions, but reached significance only in HEK293-GABA_A_R cells (RT, *P* = 0.0047; HW, *P* = 0.03; Student's paired *t*-test; Figs 7A and B).

Despite these similarities, spontaneous IPSCs were significantly larger in HEK293-GABA_A_R-NL2 cells (median, −56.6 pA; 25/75%, −44/−159 pA; *n* = 6; Fig. 6D, insert) than in HEK293-GABA_A_R cells (median, −15.3 pA; 25/75%, −13.7/−18.7 pA; *n* = 7; Fig. 4D, insert; *P* = 0.0012, Mann–Whitney *U*-test). Miniature IPSC amplitudes were also significantly larger in HEK293-GABA_A_R-NL2 cells than in HEK293-GABA_A_R cells (median, −27.2 pA; 25/75%, −25.1/−29 pA; Fig. 6D, insert; and median, −12.3 pA, 25/75%, −10.7/−15.3 pA; Fig. 4D, insert; *P* = 0.0012, Mann–Whitney *U*-test). However, the difference in mIPSC amplitude (2.2-fold) does not entirely explain the large increase in sIPSC amplitude seen when NL2 was co-expressed (3.7-fold). This may indicate that both the number of active synapses provided by each axon and the size of the response produced by each contact are larger in HEK293-GABA_A_R-NL2 cells. Thus, whereas GABA_A_Rs and NL2 can initiate contact formation independently of each other, when co-expressed they act cooperatively to promote further formation and strengthening of synaptic connections. Expressed together, they appear to increase both the quantal amplitudes and the number of active release sites provided by each axon.

## Discussion

This study demonstrates, for the first time, that the expression of GABA_A_Rs by a postsynaptic cell is sufficient to initiate the adhesion of GABAergic axons and the formation of functional synapses. When GABA_A_Rs were co-expressed with NL2 in this system, the effect on synapse formation exceeded the individual effects of these two proteins. In addition, connections with HEK293-GABA_A_R cells were strengthened when NL2 was added, suggesting a cooperative interaction.

The structural role of GABA_A_Rs in synapse formation found here, with an *in vitro* co-culture model system, is supported by results emerging from the *in vivo* analysis of mutant mice lacking specific GABA_A_R α subunits. For example, in α1 subunit knockout mice, the function and synaptic localization of gephyrin, a major postsynaptic scaffold protein, at inhibitory synapses, is disrupted (Fritschy *et al*., 2012). Similarly, in CA1 pyramidal neurones of α2 subunit knockout mice, clustering of both gephyrin and of NL2 is decreased in many subcellular compartments, but most prominently in the region of the axon initial segment, where α2 subunit-containing GABAergic synapses are abundant (Nusser *et al*., 1996; Nyiri *et al*., 2001; Panzanelli *et al*., 2011). The result presented here may also help to explain why deleting all neuroligin isoforms does not prevent synapse formation *in vivo*, but impairs their functional maturation (Varoqueaux *et al*., 2006; Poulopoulos *et al*., 2009).

The presence of gephyrin in control HEK293 cells has been reported previously, particularly in dividing cells (Wu *et al*., 2012). A role for gephyrin in the consolidation of synapse-like contacts in these co-cultures cannot therefore be excluded. That gephyrin, if present, does not initiate contact formation is indicated by the lack of contacts with control HEK293 cells, or with HEK293 cells transfected with glutamate receptors. Another postsynaptic scaffold protein, collybistin, has been shown to play an important role in the postsynaptic accumulation of GABA_A_Rs in neurones, but not to be synaptogenic (Chiou *et al*., 2011). The very high-density plasma membrane expression of GABA_A_Rs in the present HEK293-GABA_A_R cells may have removed the need for collybistin to concentrate receptors in these co-cultures, as previous studies have indicated that collybistin is not expressed in HEK293 cells (Kins *et al*., 2000).

Direct *in vivo* evidence for a role for GABA_A_Rs in synapse assembly has yet to emerge. The multiplicity of GABA_A_R subtypes expressed in neurones (Schofield *et al*., 1987; Sieghart, 2006), the vast array of presynaptic and postsynaptic proteins, in addition to the receptors, found to populate the synaptic cleft, and the possibility that removal or modification of any one building block may result either in its replacement by another or in a string of knock-on consequences, present enormous difficulties in the interpretation of any study designed to identify the unique role(s) of a specific protein. Although they are far from the situation *in vivo* and are subject to all of the caveats that should surround any study in a reduced system, these co-cultures have allowed the potential for GABA_A_Rs to participate directly in synapse formation to be demonstrated. In agreement with studies of synapse formation in NL2 knockout mice *in vivo* (Varoqueaux *et al*., 2006; Blundell *et al*., 2009; Gibson *et al*., 2009), although in contrast to the conclusions reached in relation to some of the previous co-culture studies (Scheiffele *et al*., 2000; Dean *et al*., 2003; Graf *et al*., 2004; Chih *et al*., 2005; Dong *et al*., 2007), we show that NL2 is not an absolute requirement for the formation of functional GABAergic synapses, because synapse-like contacts, capable of supporting both spontaneous ‘miniature’ synaptic events and AP-driven GABA release, were induced by GABA_A_Rs alone. The discrepancies between our study and previous *in vitro* studies could perhaps be explained, at least in part, by the different combinations of neuronal cell types and postsynaptic GABA_A_R subtypes tested. This, in addition, to the high level and consistency of cell surface expression of GABA_A_R subunits in the stably transfected HEK293 cell line used in our study, and in contrast to the transiently expressed GABA_A_Rs in previous studies, may have been crucial for the reliable detection of synapse formation and activity across the population of cells in co-culture.

The number of functional contacts was enhanced significantly by concomitant overexpression of NL2, as seen in neurones (Fu & Vicini, 2009). Stable connections, involving several synapse-like contacts per axon, do occur in the absence of NL2. However, comparison of sIPSC, AP-IPSC and mIPSC amplitudes indicates that single axon connections may involve more presynaptic terminals, and that each terminal elicits a stronger postsynaptic response when NL2 is co-expressed together with GABA_A_Rs. NL2 may also be important for the rigid membrane appositions typical of synapses *in situ*, and contribute to the stabilization of contacts (compare Fig. 3D and Figs S2A and B), and may increase the number of synapses formed by each axon, thereby playing an essential role in normal synaptic activity. However, NL2 does not appear to be essential for GABAergic synapse formation, either *in vivo* (Varoqueaux *et al*., 2006; Blundell *et al*., 2009; Gibson *et al*., 2009) or *in vitro*.

That these α1/β2/γ2-GABA_A_Rs were sufficient alone to support and stabilize functional synapse-like contacts is interesting in the light of a study by Gibson *et al*. (2009). In this study, the synapses innervated by fast-spiking, parvalbumin-containing interneurones in the hippocampus, which are mediated by α1-GABA_A_Rs (Thomson *et al*., 2000; Nyiri *et al*., 2001), were found to be the most powerfully affected in NL2 knockouts. Both quantal amplitude and quantal content (i.e. the number of quanta, or synapses, contributing to each event) were lower than at wild-type connections. These findings in NL2 knockout mice have a striking parallel in the present study, where the absence of NL2 coincided with decreases in both the number of functional synapses and the quantal amplitude, in a much more reduced system employing a different class of presynaptic neurone.

A larger mIPSC, or quantal amplitude, is typically explained either by a larger number of postsynaptic receptors, or by an increase in their single channel conductance. HEK293-GABA_A_R-NL2 cells received a large number of synapse-like contacts, which were often very close neighbours (Fig. 5A; Fig. S2A), whereas HEK293-GABA_A_R cells received more sparse innervation (Figs 3A and D). If such a finding were obtained in a neuronal system, it might suggest that the larger quantal amplitudes seen in HEK293-GABA_A_R-NL2 cells are attributable to spill-over from one terminal to receptors lying under one or more neighbouring terminals. However, although these cultures did not contain glial cells, whose active re-uptake of GABA might otherwise have curtailed its diffusion, the extracellular space in the co-cultures is very large, and the released GABA can be expected to have diffused rapidly away from the HEK293 cell. There was, moreover, little evidence for clustering of receptors in these HEK293-GABA_A_R cells (Fig. 1C; Fig. S1A), and there was no evidence that the surface expression of GABA_A_Rs differed between HEK293-GABA_A_R and HEK293-GABA_A_R-NL2 cells. Spread of GABA to a larger, more widely distributed population of receptors is therefore not an adequate explanation for the larger quantal amplitudes seen here with NL2 overexpression.

How, then, might overexpression of NL2 enhance the activity of GABA_A_Rs in this system? An economical, currently not easily refutable hypothesis, for which there is at present only indicative evidence, is suggested by a recent finding (Zhang *et al*., 2010). Neurexin-2β, either overexpressed in the postsynaptic cell or applied to the culture as free protein, decreased GABA currents via direct interaction with α1-GABA_A_Rs, in a neuroligin-independent manner. It is possible that, in the absence of NL2, neurexin-2β interacts with GABA_A_Rs in a way that promotes adhesion but suppresses receptor activity, and that this suppression is relieved or reduced when NL2 is also present. During synapse formation, an interaction between the appropriate presynaptic neurexin and postsynaptic GABA_A_R might be sufficient to establish a synaptic connection. This connection would then become stronger as NL2 became colocalized with the receptors, and the interaction between receptor and neurexin was thereby modified. *In vivo*, NL2 would then recruit further postsynaptic density proteins (Poulopoulos *et al*., 2009), and perhaps this, together with the increased synaptic activity, would further strengthen and stabilize the connection (Hartman *et al*., 2006; Varoqueaux *et al*., 2006; Chubykin *et al*., 2007). As a very reduced system was used for this study, it is not possible to propose that complex developments at the postsynaptic density could have initiated an increase in the stability or density of the innervation here. The most likely explanation for this is therefore that the enhanced synaptic activity, resulting from the larger quantal amplitudes in HEK293-GABA_A_R-NL2 cells, contributes to the increase in the density of innervation. Although the proposed modulatory role of neurexins and NL2 in synapse formation initiated by GABA_A_Rs awaits further investigation, the co-culture system described here may be particularly advantageous in these experiments, as it provides tight control of expression and precise molecular manipulation of component players, both individually and in combination.

In conclusion, using a multidisciplinary approach, we have demonstrated that functional synapses can form in the absence of neuronal trans-synaptic adhesion molecules, if GABA_A_Rs are present. By promoting the adhesion of inhibitory axon terminals and their stabilization, GABA_A_Rs may play an important role in mechanisms underlying the development of inhibitory synapses.

## Abbreviations

AP: action potential
AP-IPSC: action potential-driven inhibitory postsynaptic current
d-AP5: d-2-amino-5-phosphonovalerate
DNQX: 6,7-dinitroquinoxaline-2,3-dione
GABA_A_R: GABA type A receptor
GAD: glutamic acid decarboxylase
HEK293: human embryonic kidney 293
HRP: horseradish peroxidase
HW: width at half amplitude
IPSC: inhibitory postsynaptic current
mIPSC: miniature inhibitory postsynaptic current
NL2: neuroligin-2
NMDA: *N*-methyl-d-aspartate
PBS: phosphate-buffered saline
PFA: paraformaldehyde
RT: 10–90% rise time
SDTC: standard deviation time course
SEM: standard error of the mean
sIPSC: spontaneous inhibitory postsynaptic current
TTX: tetrodotoxin citrate
YFP: yellow fluorescent protein

## Supporting Information

**Fig. S1**. Innervation of HEK293-GABAAR-NL2 cells by embryonic basal ganglia GABAergic medium spiny neurones in co-culture. **Fig. S2**. Innervation of HEK293-GABAAR-NL2 and HEK293 cells by medium spiny neurones at the EM level. **Movie S1.** GABAergic medium spiny neurones and HEK293-GABAAR cells in co-culture form stable contacts.

## References

[b1] Blundell J, Tabuchi K, Bolliger MF, Blaiss CA, Brose N, Liu X, Sudhof TC, Powell CM (2009). Increased anxiety-like behavior in mice lacking the inhibitory synapse cell adhesion molecule neuroligin 2. Genes Brain Behav.

[b2] Buerli T, Pellegrino C, Baer K, Lardi-Studler B, Chudotvorova I, Fritschy JM, Medina I, Fuhrer C (2007). Efficient transfection of DNA or shRNA vectors into neurons using magnetofection. Nat. Protoc.

[b3] Chazot PL, Coleman SK, Cik M, Stephenson FA (1994). Molecular characterization of N-methyl-D-aspartate receptors expressed in mammalian cells yields evidence for the coexistence of three subunit types within a discrete receptor molecule. J. Biol. Chem.

[b4] Chih B, Engelman H, Scheiffele P (2005). Control of excitatory and inhibitory synapse formation by neuroligins. Science.

[b5] Chiou TT, Bonhomme B, Jin H, Miralles CP, Xiao H, Fu Z, Harvey RJ, Harvey K, Vicini S, De Blas AL (2011). Differential regulation of the postsynaptic clustering of gamma-aminobutyric acid type A (GABAA) receptors by collybistin isoforms. J. Biol. Chem.

[b6] Chubykin AA, Atasoy D, Etherton MR, Brose N, Kavalali ET, Gibson JR, Sudhof TC (2007). Activity-dependent validation of excitatory versus inhibitory synapses by neuroligin-1 versus neuroligin-2. Neuron.

[b7] Dean C, Scholl FG, Choih J, DeMaria S, Berger J, Isacoff E, Scheiffele P (2003). Neurexin mediates the assembly of presynaptic terminals. Nat. Neurosci.

[b8] Dong N, Qi J, Chen G (2007). Molecular reconstitution of functional GABAergic synapses with expression of neuroligin-2 and GABAA receptors. Mol. Cell. Neurosci.

[b9] Farrant M, Kaila K (2007). The cellular, molecular and ionic basis of GABA(A) receptor signalling. Prog. Brain Res.

[b10] Fernandez-Alfonso T, Kwan R, Ryan TA (2006). Synaptic vesicles interchange their membrane proteins with a large surface reservoir during recycling. Neuron.

[b11] Fiala JC (2005). Reconstruct: a free editor for serial section microscopy. J. Microsc.

[b12] Fritschy JM, Mohler H (1995). GABAA-receptor heterogeneity in the adult rat brain: differential regional and cellular distribution of seven major subunits. J. Comp. Neurol.

[b13] Fritschy JM, Panzanelli P, Tyagarajan SK (2012). Molecular and functional heterogeneity of GABAergic synapses. Cell. Mol. Life Sci.

[b14] Fu Z, Vicini S (2009). Neuroligin-2 accelerates GABAergic synapse maturation in cerebellar granule cells. Mol. Cell. Neurosci.

[b15] Fujiyama F, Fritschy JM, Stephenson FA, Bolam JP (2000). Synaptic localization of GABA(A) receptor subunits in the striatum of the rat. J. Comp. Neurol.

[b16] Futai K, Doty CD, Baek B, Ryu J, Sheng M (2013). Specific trans-synaptic interaction with inhibitory interneuronal neurexin underlies differential ability of neuroligins to induce functional inhibitory synapses. J. Neurosci.

[b17] Gibson JR, Huber KM, Sudhof TC (2009). Neuroligin-2 deletion selectively decreases inhibitory synaptic transmission originating from fast-spiking but not from somatostatin-positive interneurons. J. Neurosci.

[b18] Goffin D, Ali AB, Rampersaud N, Harkavyi A, Fuchs C, Whitton PS, Nairn AC, Jovanovic JN (2010). Dopamine-dependent tuning of striatal inhibitory synaptogenesis. J. Neurosci.

[b19] Graf ER, Zhang X, Jin SX, Linhoff MW, Craig AM (2004). Neurexins induce differentiation of GABA and glutamate postsynaptic specializations via neuroligins. Cell.

[b20] Gross A, Sims RE, Swinny JD, Sieghart W, Bolam JP, Stanford IM (2011). Differential localization of GABA(A) receptor subunits in relation to rat striatopallidal and pallidopallidal synapses. Eur. J. Neurosci.

[b21] Hartman KN, Pal SK, Burrone J, Murthy VN (2006). Activity-dependent regulation of inhibitory synaptic transmission in hippocampal neurons. Nat. Neurosci.

[b22] Kang Y, Zhang X, Dobie F, Wu H, Craig AM (2008). Induction of GABAergic postsynaptic differentiation by alpha-neurexins. J. Biol. Chem.

[b23] Kins S, Betz H, Kirsch J (2000). Collybistin, a newly identified brain-specific GEF, induces submembrane clustering of gephyrin. Nat. Neurosci.

[b24] Missler M, Zhang W, Rohlmann A, Kattenstroth G, Hammer RE, Gottmann K, Sudhof TC (2003). Alpha-neurexins couple Ca^2+^ channels to synaptic vesicle exocytosis. Nature.

[b25] Nusser Z, Sieghart W, Benke D, Fritschy JM, Somogyi P (1996). Differential synaptic localization of two major gamma-aminobutyric acid type A receptor alpha subunits on hippocampal pyramidal cells. Proc. Natl. Acad. Sci. USA.

[b26] Nyiri G, Freund TF, Somogyi P (2001). Input-dependent synaptic targeting of alpha(2)-subunit-containing GABA(A) receptors in synapses of hippocampal pyramidal cells of the rat. Eur. J. Neurosci.

[b27] Panzanelli P, Gunn BG, Schlatter MC, Benke D, Tyagarajan SK, Scheiffele P, Belelli D, Lambert JJ, Rudolph U, Fritschy JM (2011). Distinct mechanisms regulate GABAA receptor and gephyrin clustering at perisomatic and axo-axonic synapses on CA1 pyramidal cells. J. Physiol.

[b28] Poulopoulos A, Aramuni G, Meyer G, Soykan T, Hoon M, Papadopoulos T, Zhang M, Paarmann I, Fuchs C, Harvey K, Jedlicka P, Schwarzacher SW, Betz H, Harvey RJ, Brose N, Zhang W, Varoqueaux F (2009). Neuroligin 2 drives postsynaptic assembly at perisomatic inhibitory synapses through gephyrin and collybistin. Neuron.

[b29] Scheiffele P, Fan J, Choih J, Fetter R, Serafini T (2000). Neuroligin expressed in nonneuronal cells triggers presynaptic development in contacting axons. Cell.

[b30] Schofield PR, Darlison MG, Fujita N, Burt DR, Stephenson FA, Rodriguez H, Rhee LM, Ramachandran J, Reale V, Glencorse TA (1987). Sequence and functional expression of the GABA A receptor shows a ligand-gated receptor super-family. Nature.

[b31] Shen K, Scheiffele P (2010). Genetics and cell biology of building specific synaptic connectivity. Annu. Rev. Neurosci.

[b32] Siddiqui TJ, Craig AM (2011). Synaptic organizing complexes. Curr. Opin. Neurobiol.

[b33] Sieghart W (2006). Structure, pharmacology, and function of GABAA receptor subtypes. Adv. Pharmacol.

[b34] Sudhof TC (2008). Neuroligins and neurexins link synaptic function to cognitive disease. Nature.

[b35] Thomson AM, Bannister AP, Hughes DI, Pawelzik H (2000). Differential sensitivity to Zolpidem of IPSPs activated by morphologically identified CA1 interneurons in slices of rat hippocampus. Eur. J. Neurosci.

[b36] Varoqueaux F, Aramuni G, Rawson RL, Mohrmann R, Missler M, Gottmann K, Zhang W, Sudhof TC, Brose N (2006). Neuroligins determine synapse maturation and function. Neuron.

[b37] Ventimiglia R, Lindsay RM, Goslin K, Banker G (1998). Rat striatal neurons in low-density, serum-free culture. Culturing Nerve Cells.

[b38] Wu X, Wu Z, Ning G, Guo Y, Ali R, Macdonald RL, De Blas AL, Luscher B, Chen G (2012). gamma-Aminobutyric acid type A (GABAA) receptor alpha subunits play a direct role in synaptic versus extrasynaptic targeting. J. Biol. Chem.

[b40] Zhang C, Atasoy D, Arac D, Yang X, Fucillo MV, Robison AJ, Ko J, Brunger AT, Sudhof TC (2010). Neurexins physically and functionally interact with GABA(A) receptors. Neuron.

[b39] Zhang W, Rohlmann A, Sargsyan V, Aramuni G, Hammer RE, Sudhof TC, Missler M (2005). Extracellular domains of alpha-neurexins participate in regulating synaptic transmission by selectively affecting N- and P/Q-type Ca^2+^ channels. J. Neurosci.

