# Supplementary material for: GABAA receptors can initiate the formation of functional inhibitory GABAergic synapses
Source: Eur J Neurosci. 2013 Aug 5;38(8):3146–58. doi: 10.1111/ejn.12331 (PMC4028986; doi:10.1111/ejn.12331)
Supplement: Supplementary file 1 — Fig. S1. Innervation of HEK293-GABAAR-NL2 cells by embryonic basal ganglia GABAergic medium spiny neurones in co-culture. Fig. S2. Innervation of HEK293-GABAAR-NL2 and HEK293 cells by medium spiny neurones at the EM level. Movie S1. GABAergic medium spiny neurones and HEK293-GABAAR cells in co-culture form stable contacts. [file ejn0038-3146-sd1.pdf]

SUPPORTING INFORMATION

Supplementary Movie. GABAergic medium spiny neurones and HEK293-GABA$_A$R cells in co-culture form stable contacts. Time lapse movie of contacts between neurones expressing GFP (green) and HEK293-GABA$_A$R cells expressing p-Cherry (red) formed 24 h after plating. Two channels were monitored simultaneously for 120 min. Images were taken at 1-minute intervals. Scale bar = 20 µm.

Supplementary Figure 1. Innervation of HEK293-GABA$_A$R-NL2 by embryonic basal ganglia GABAergic medium spiny neurones in co-culture. (A) Immunolabelling of synapse-like contacts between GAD65 positive terminals of GABAergic neurones (green) and postsynaptic NL2-Cherry- (red) and µ2 GABA$_A$R subunit- (blue) labelled HEK293-GABA$_A$R-NL2 cells. Scale bar = 10 µm. (B) Recordings of spontaneous postsynaptic currents (sIPSCs) in a HEK293-GABA$_A$R-NL2 cell (upper trace), and, following TTX application (1 µM), of miniature postsynaptic currents (mIPSCs) in the absence (middle) or presence of bicuculline (10 µM, lower trace). (C) Time course of putative contact formation. (D) Proportion of HEK293-GABA$_A$R-NL2 cells displaying sIPSCs increases with time in co-culture to 24 h.

Supplementary Figure 2. Innervation of HEK293-GABA$_A$R-NL2 and HEK293-NL2 cells by medium spiny neurones at the EM level. (A) Ultrastructure of synaptic contacts with a HEK293-GABA$_A$R-NL2 cell (top panel scale = 10 µm, two middle panels scale = 5 µm, two lower panels scale = 1 µm), or (B) with a HEK293-NL2 cell (top panel and middle panel

scale = 5 µm, four lower panels scale = 0.5 µm). The regions selected for ultrastructural analysis are indicated in the low power images of the selected HEK293 cells in the top panels (of A and B) and in a low power EM in middle panels.

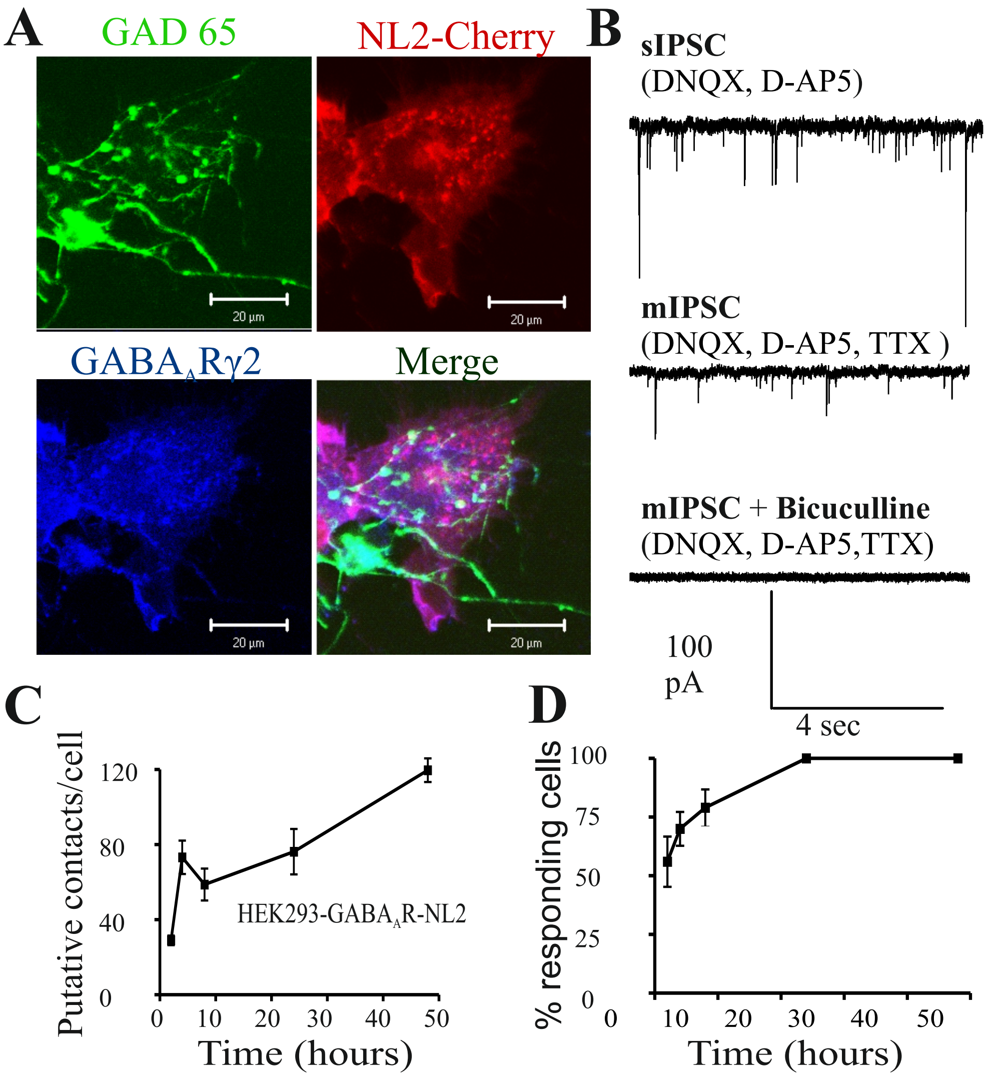

Supplementary Figure 1

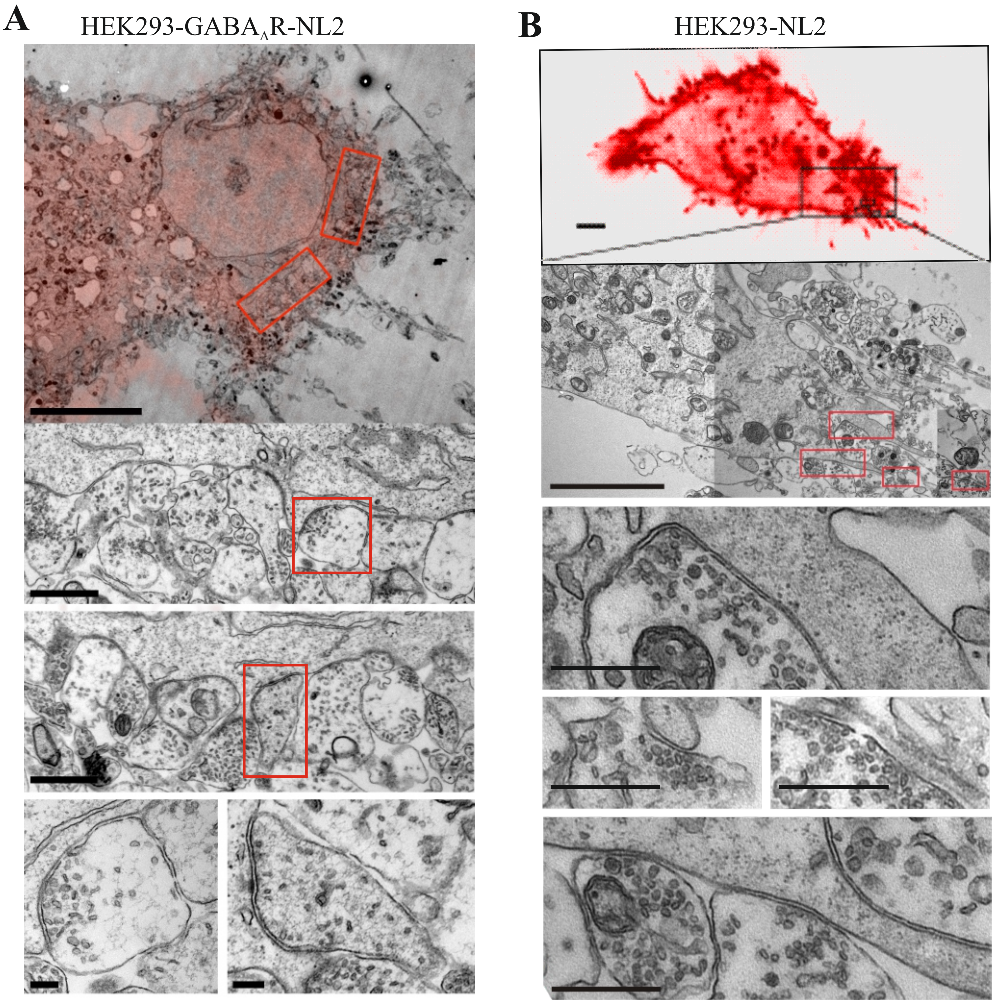

Supplementary Figure 2
